# The Jaws and Teeth in Semi-Barbarous Races of Men

**Published:** 1852-10

**Authors:** 


### ARTICLE XVII.

### *The Jaws and Teeth in Semi-Barbarous Races of Men.*

Mr. Levison, a very able and scientific writer upon this subject, observes in the *Lancet,* that in submitting some important facts resulting from dental irritation in the organs of the mouth generally, and also some cases of the disturbance of remote organs, the effect of reflex-nervous action, permit me to call attention, in this paper, by the way of introduction, to what appears to be one of the penalties consequent on a state of civilization—namely, that the jaws of civilized man are more contracted than the aborigines of different countries usually spoken of as barbarous, or semi-barbarous; that in civilized nations, the physiognomy is well marked; that the contraction of the jaws causes the permanent teeth to be either jammed together, or else there are induced various kinds of malformation: and in both instances, there exists a predisposition for the teeth to become carious, or remote organs are implicated, often inducing much functional disturbance in the brain, spinalis, and the chylopoietic viscera.

It will therefore be important to specify some few preliminary facts. The jaws of the African races, the South Sea Islanders, and some other barbarous tribes, form a half ellipsis; the lower, at its anterior portion, being a smaller curve than the upper, so that although the molars meet at their grinding surfaces when the jaws close together, yet the upper front teeth (including the incisors and cuspidati) shut over those beneath, and act as a pair of shears; and as a first process cutting the food into small pieces, prior to being conveyed to the grinding operation of the molar teeth. In the jaws of any of the races mentioned, (unless injured by their civilized brethren,) there are usually thirty-two teeth (the permanent set) arranged in a very symmetrical manner, giving ample space for each tooth, and thus preventing the dentes sapientiæ from being jammed against the coronoid processes in the lower, or forced too near to the glenoid cavities of the upper jaw: whilst in the mouths of the most civilized people, the anterior portions of both are so contracted, as rarely to admit the proper arrangement of the incisors and cuspidati, without extracting the first bicuspid on both sides, and in each jaw. And so well marked are these peculiarities, that in a paper I read, "On the causes for the decay of teeth in civilized communities," to the members of the Brighton Royal Scientific and Literary Institution, I ventured to state, "that if a number of national crania were placed promiscuously on a table, I would arrange them ethnologically according to their comparative degrees of civilization, merely by the form of their jaws and the position of their teeth."

It may be asked whether these differences can be satisfactorily explained? Are they the result of some positive laws, or merely isolated and accidental consequences? That I may not appear presumptuous in attempting to answer these questions, I may premise that my experience is deduced from nearly thirty years' attention to the subject, and from numerous facts which I have collected. The following inferences seem to be correct:—That the difference in the jaws of barbaric races and those of civilized nations, is caused by the observance or non-observance of the physical and organic laws during the period of the growth and development of the bodily organs; that the races of men with ample jaws and finely-formed teeth had lived the greater part of their infancy, childhood, and youth in the open air; that they had freely exercised their limbs, and partook of a simple diet; and that their brains had not been fatigued and annoyed with lessons in crowded school-rooms during their early days; and when taken out for a walk, they were not moved by a drill-master, but were allowed to act from the impulses of nature; that, in consequence of this sort of physical training, they had healthy appetites, and needed neither condiments nor stimulants. Head-aches and languor they knew not, and when the evening sun declined below the western horizon, their previous fatigue, procured for them healthy and refreshing sleep. In consequence of such obedience to the natural laws, their secretions were natural without being drugged with calomel. They had therefore neither rickets nor distorted spines; hence all their bodily functions were normal, and the organs of the chest, the abdomen, and the mouth, were in consequence amply developed. And just because the converse of all this takes place in what is called "a state of civilization," the general stamina of the young is injured—their stomachs deranged—their brains rendered too irritable, inducing many affections, sometimes immediate and sometimes latent; and the mouth also becomes more or less implicated.

If the days of childhood were devoted to the goddess Hygeia, and the perceptive faculties allowed to exercise themselves by observing the wonders of art and the beauties of nature—then civilization would bring to all positive blessings, and a higher advantage co-existing with rude good health. It is true, that if these views were generally entertained, there would be less to do for dentists, of whom the saying which George Colman applied to authors, may be said of them,

"*They wriggle along in shoals;*"

and they might exclaim, if our suggestions were practiced,

"Othello's occupation's gone!"

but the world would be spared much suffering and vast inconvenience. Physical health is one of the greatest sources of happiness to the child. Its value may be known by watching the gambols of well-formed children, with cheeks in which the rose and the lily blend most harmoniously, giving brightness to their laughing, happy-looking eyes, as they throw their pliant bodies and vigorous limbs into the most graceful attitudes, whilst bounding over the green-sward, like young gazelles, positively intoxicated with the joys of mere physical existence! Or watch them pursuing with agility the fleeting butterfly, or gathering wild flowers: every sense hath its full measure of enjoyment, and the stock of health thus daily laying up, is a treasury of inexhaustible pleasure. Nor is this healthy condition of the body confined to the organs of motion, to the digestive organs, and to the mouth; but the brain, so essential for every mental operation, participates in this paramount advantage. The latter important organ, (the brain,) being supplied with healthy and well-oxygenized blood, its quality is improved; its susceptibility is greater; its powers of endurance regular; its quickness of perception keener; and its impressions more lasting. So that, for all purposes, attention to physical development is proved to be most thrifty; for experience confirms the lessons which nature renders so palpable; and if, in spite of all the warnings of ill-formed, contracted mouths, weak digestions, feeble bodies, irritable brain and nervous systems, we still force children to taste too early "the tree of knowledge," we must in every instance bear the certain "pains and penalties" for our contumany.

